# Hematuria, Proteinuria, and Acute Kidney Injury in a Patient Presenting with Abdominal Pain

**DOI:** 10.34067/KID.0000001073

**Published:** 2026-05-28

**Authors:** Inês Alexandre, Rita Theias, Patrícia Carrilho

**Affiliations:** 1 Department of Nephrology, Hospital Prof. Dr. Fernando Fonseca, Lisboa, Portugal; 2 Department of Pathology, Hospital Prof. Dr. Fernando Fonseca, Lisboa, Portugal

**Keywords:** AKI, ANCA, GN, renal biopsy

## Abstract

**Figure:**
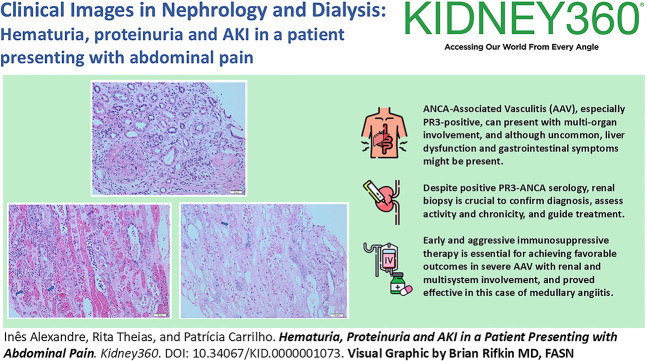

## Case Presentation

A 67-year-old woman presented to the emergency room with fatigue, anorexia, nausea, and abdominal right upper-quadrant pain with 1 month evolution. Medical history included asthma, treated with bronchodilators. At admission, she had no remarkable signs on physical examination apart from hypertension and mild abdominal pain. Serum creatinine (sCr) was 0.92 mg/dl, urea 24.8 mg/dl, but urinalysis presented hemoglobin (3+), leukocytes (15/hpf), and proteinuria quantified by a urinary albumin-to-creatinine ratio of 478 mg/g. In addition, gamma-glutamyl transferase and alkaline phosphatase levels were mildly increased (360 and 164 U/L, respectively).

**Figure 1:**
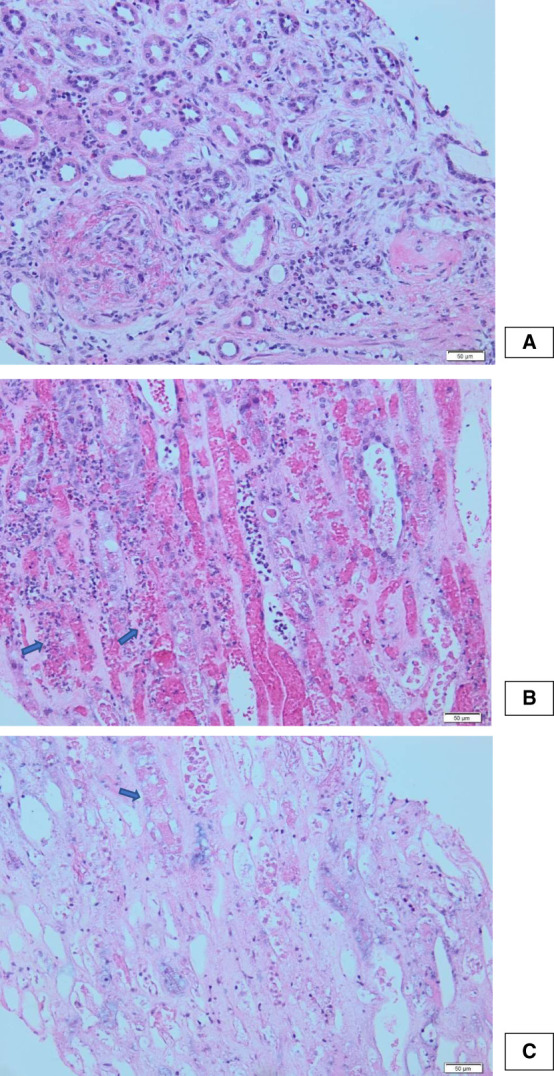
**Renal biopsy histology.** (A) Cortical region with a glomerulus with a cellular crescent and fibrinoid necrosis. (B) Medullary region with congestive vasa recta and abundant neutrophils and erythrocyte extravasation in perivascular stroma nuclear dust associated. (C) Medullary region with extensive ischemic necrosis.

The patient was admitted for investigation of abdominal symptoms; however, ultrasound was unremarkable. In the first days of hospitalization, she developed AKI, with sCr raising up to 7.5 mg/dl in 6 days and urea to 157 mg/dl. Persistent leukocytes and red blood cells were observed on urinalysis. Renal ultrasound showed bilateral normal kidney size with preserved differentiation. Urinary tract obstruction and nephrolithiasis were ruled out. The etiological investigation revealed a positive cytoplasmic ANCA, with a PR3 of 237 U/ml, and negative results for the remaining immunologic, infectious, and monoclonal gammopathy screenings. Renal biopsy yielded two macroscopically hemorrhagic samples. She was started on plasma exchange, methylprednisolone pulses, and combination therapy with rituximab and cyclophosphamide. Histologic analysis revealed a pauci-immune crescentic GN with 20% crescents showing fibrinoid necrosis (Figure 1A), severe medullary angiitis (Figure 1B) with extensive necrosis (Figure 1C), and no signs of chronicity.

She completed five sessions of plasma exchange every other day, two administrations of rituximab, and one of cyclophosphamide while hospitalized with renal function improvement to sCr 2.75 mg/dl and urea 108 mg/dl. Notably, after immunosuppression initiation, constitutional and gastrointestinal symptoms, as well as laboratory liver enzymes elevation, resolved in a few days.

The patient completed induction treatment with five additional outpatient cyclophosphamide cycles. Post-treatment laboratory test results showed sCr 1.82 mg/dl, urea 81 mg/dl, with reduction of hematuria (+1), urinary albumin-to-creatinine ratio 0.091 g/g, and PR3-ANCA 2.3 U/ml.

## Discussion

ANCA-associated vasculitis (AAV) is the most common cause of rapidly progressive GN and constitutional symptoms may be present for several months before diagnosis.^1^ Liver involvement is not a common manifestation, but it can occur, especially in granulomatosis with polyangiitis.^2^

Regarding renal disease, this patient exhibited a rare pathologic variant, medullary angiitis. This is characterized by interstitial hemorrhage and necrosis in the renal medulla, accompanied by PMN infiltration, as seen in this biopsy. Medullary angiitis typically presents with severe renal impairment and multisystem disease. In this case, the hemorrhagic biopsy samples and marked hematuria should also raise suspicion for this diagnosis.^3^

Early combined immunosuppression with plasma exchange, rituximab, and cyclophosphamide proved to be a safe and effective approach for inducing remission.

## Teaching Points

- AAV, especially PR3-positive, can present with multiorgan involvement, and although uncommon, liver dysfunction and gastrointestinal symptoms might be present.
- Despite positive PR3-ANCA serology, renal biopsy is crucial to confirm diagnosis, assess activity and chronicity, and guide treatment.
- Early and aggressive immunosuppressive therapy is essential for achieving favorable outcomes in severe AAV with renal and multisystem involvement and proved effective in this case of medullary angiitis.

## Supplementary Material
