# Supplementary material for: Hematuria, Proteinuria, and Acute Kidney Injury in a Patient Presenting with Abdominal Pain
Source: Kidney360. 2026 May 28;7(5):1189–91. doi: 10.34067/KID.0000001073 (PMC13229434; doi:10.34067/KID.0000001073)
Supplement: Supplementary file 1 [file kidney360-7-1189-s001.pdf]

ASN American Society of Nephrology
JASN Journal of the American Society of Nephrology
KIDNEY360 Accessing Our World From Every Angle
CJASN Clinical Journal of the American Society of Nephrology

ASN Journal Disclosure Form

As per ASN journal policy, I have disclosed any financial relationships or commitments I have held in the past 36 months as included below. I have listed my Current Employer below to indicate there is a relationship requiring disclosure. If no relationship exists, my Current Employer is not listed.

I. Alexandre reports the following:
Employer: Hospital Prof Dr Fernando Fonseca

I understand that the information above will be published within the journal article, if accepted, and that failure to comply and/or to accurately and completely report the potential financial conflicts of interest could lead to the following: 1) Prior to publication, article rejection, or 2) Post-publication, sanctions ranging from, but not limited to, issuing a correction, reporting the inaccurate information to the authors' institution, banning authors from submitting work to ASN journals for varying lengths of time, and/or retraction of the published work.

Name: Ines Alexandre
Manuscript ID: K360-2025-001228R1
Manuscript Title: Hematuria, proteinuria and AKI in a patient presenting with abdominal pain
Date of Completion: November 2, 2025
Disclosure Updated Date: November 2, 2025

ASN American Society of Nephrology
JASN Journal of the American Society of Nephrology
KIDNEY360 Accessing Our World From Every Angle
CJASN Clinical Journal of the American Society of Nephrology

ASN Journal Disclosure Form

As per ASN journal policy, I have disclosed any financial relationships or commitments I have held in the past 36 months as included below. I have listed my Current Employer below to indicate there is a relationship requiring disclosure. If no relationship exists, my Current Employer is not listed.

P. Carrilho reports the following:
Employer: hospital professor doutor fernando fonseca; and Honoraria: Fresenius Medical Care.

I understand that the information above will be published within the journal article, if accepted, and that failure to comply and/or to accurately and completely report the potential financial conflicts of interest could lead to the following: 1) Prior to publication, article rejection, or 2) Post-publication, sanctions ranging from, but not limited to, issuing a correction, reporting the inaccurate information to the authors' institution, banning authors from submitting work to ASN journals for varying lengths of time, and/or retraction of the published work.

Name: Patricia Carrilho
Manuscript ID: K360-2025-001228R2
Manuscript Title: Hematuria, proteinuria and AKI in a patient presenting with abdominal pain
Date of Completion: November 10, 2025
Disclosure Updated Date: November 10, 2025

ASN
American Society of Nephrology
JASN
Journal of the American Society of Nephrology
KIDNEY360
Accessing Our World From Every Angle
CJASN
Clinical Journal of the American Society of Nephrology

ASN Journal Disclosure Form

As per ASN journal policy, I have disclosed any financial relationships or commitments I have held in the past 36 months as included below. I have listed my Current Employer below to indicate there is a relationship requiring disclosure. If no relationship exists, my Current Employer is not listed.

R. Theias Manso reports the following:
Employer: Hospital Prof. Doutor Fernando Fonseca

I understand that the information above will be published within the journal article, if accepted, and that failure to comply and/or to accurately and completely report the potential financial conflicts of interest could lead to the following: 1) Prior to publication, article rejection, or 2) Post-publication, sanctions ranging from, but not limited to, issuing a correction, reporting the inaccurate information to the authors' institution, banning authors from submitting work to ASN journals for varying lengths of time, and/or retraction of the published work.

Name: Rita Theias Manso
Manuscript ID: K360-2025-001228R1
Manuscript Title: Hematuria, proteinuria and AKI in a patient presenting with abdominal pain
Date of Completion: December 9, 2025
Disclosure Updated Date: December 9, 2025
